# SCOUT® Radar Localisation for Nonpalpable Breast Lesions: A North Queensland Perspective

**DOI:** 10.7759/cureus.103699

**Published:** 2026-02-16

**Authors:** Tamara Fisher, William Swee Keong Khoo, Aimee Lee Jones, Alec Winder, April Miu, John Avramovic

**Affiliations:** 1 Department of General Surgery, Townsville University Hospital, Townsville, AUS; 2 Department of General Surgery, North Queensland Minimally Invasive Surgery, Townsville, AUS; 3 School of Medicine, James Cook University, Townsville, AUS

**Keywords:** breast cancer, excisional biopsy, radar localisation, scout, wide local excision

## Abstract

Introduction

Wire localisation of nonpalpable breast lesions is common. However, the logistical limitations of wire localisation have led to the development of wireless methods such as the SAVI SCOUT® system (Merit Medical Systems, South Jordan, UT, USA).

Methods

This retrospective study analysed consecutive patients undergoing SCOUT®-guided excision of nonpalpable breast lesions between February 2024 and June 2025 from one high-volume tertiary institution and one private surgical practice in North Queensland. The primary outcomes were successful reflector placement, intraoperative localisation, reflector retrieval, and positive margin rates. Secondary outcomes included time from SCOUT® placement to surgery, operative duration, intraoperative re-excision, specimen weight and volume, post-operative complications, and rates of close margins and re-excisions.

Results

A total of 170 nonpalpable breast lesions were excised from 163 patients, of which 24 patients had their lesions excised post-neoadjuvant chemotherapy (NACT). We observed 97% successful placement of the reflector, 98% successful intraoperative localisation, and 100% successful reflector retrieval. Median time from reflector insertion to surgery was six days (IQR 4, 13), with the longest duration between reflector placement and surgery being 198 days in a patient whose reflector was placed prior to commencing NACT. The mean operating time was 87.5 minutes (SD 43.5). Post-operative complications were observed in 14 patients (8.2%). Rates of involved margins and re-excisions were 9% and 19%, respectively.

Conclusion

SCOUT® is an effective and reliable method of wireless localisation for the excision of nonpalpable breast lesions.

## Introduction

Breast cancer is the most commonly diagnosed cancer in Australian women. While breast cancer incidence continues to increase, survival rates are also on the rise with the 5-year survival improving from 75% in 1987-1991 to 93% in 2017-2021 [1]. This is largely attributed to the widespread availability of screening mammography through BreastScreen, allowing for earlier diagnosis, thereby enabling the detection of lesions which may be clinically occult (nonpalpable) [2-4]. Many patients with nonpalpable breast lesions may be candidates for breast conservation. Wide local excision with adjuvant radiotherapy has been shown to be equivalent to mastectomy in survival [5] while offering improved quality of life due to more favourable cosmesis and subsequent improved psychosocial wellbeing [6-7]. Accurate pre-operative localisation is imperative for successful wide local excision.

Wire localisation of nonpalpable breast lesions has been the mainstay of localisation modalities for several decades since its introduction in the 1970s [8-10]. It involves the percutaneous insertion of a hooked wire into the lesion under ultrasound or stereotactic guidance. While it is an accurate method of localisation and relatively cost-effective, the wires can migrate or be transected. It can also cause discomfort and distress to the patient as the wire protrudes from the skin. They must be inserted on the day of surgery which can interfere with workflow often causing costly delays. These limitations have led to the development of wireless localisation techniques [11].

The SAVI SCOUT® system (Merit Medical Systems, South Jordan, UT, USA) is a wireless localisation technology that involves the implantation of a radar reflector into the breast lesion. Initial feasibility studies in the United States of America showed SCOUT® to be a safe and effective alternative to wire localisation in guiding the excision of nonpalpable breast lesions [12-14]. Few Australian hospitals have adopted the routine use of SCOUT®, and accordingly, the Australian literature is sparse. This study evaluated the placement, localisation and accurate surgical excision of nonpalpable breast lesions localised with SCOUT® in Townsville, North Queensland.

## Materials and methods

Patients

Retrospective data was manually extracted from electronic medical records from one high-volume tertiary institution and one private surgical practice in North Queensland. All female patients aged 18 years and older who underwent a SCOUT® localised excision of a nonpalpable breast lesion between February 2024 and June 2025 were included. Patients were excluded if they required dual localisation with wire and SCOUT®, or if multiple SCOUT®s were used to ‘bracket’ the lesion. Patients who received neoadjuvant chemotherapy (NACT) were included in the main cohort and also analysed separately as a subgroup. All patients with malignant pathology were discussed in a multidisciplinary team meeting attended by breast surgeons, radiologists, medical oncologists, radiation oncologists, pathologists, and breast care nurses.

Procedure

**Figure 1:**
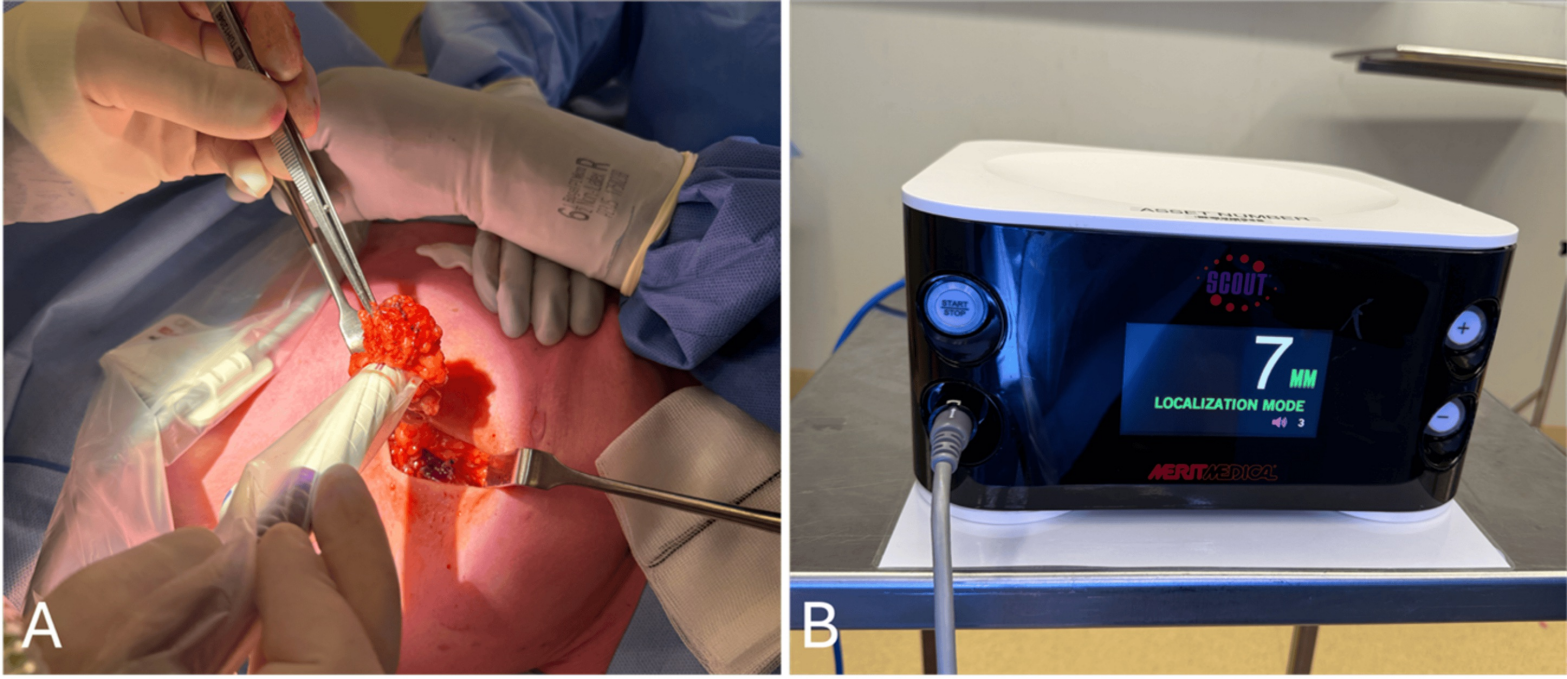
Utilisation of the SCOUT® system intraoperatively. (A) SCOUT® handpiece used to detect reflector to guide excision; (B) SCOUT® console

The SCOUT® system [15] is a wireless localisation technology that involves the implantation of a 1.2cm radar reflector into the breast lesion. The reflector consists of a light receptor, transistor switch, and two antennae that secure the reflector to the breast tissue. It is delivered via a pre-loaded 16-gauge needle under ultrasound or stereotactic guidance. In surgery, the surgeon uses a handpiece that emits pulses of infrared light and radar into the breast tissue. The reflector then transmits a radar signal back to the handpiece to provide direction and proximity to the reflector and lesion with an accuracy of within 1mm. The console displays the distance between the handpiece and reflector on its screen in real-time while generating an audible signal that increases in cadence as the handpiece moves closer to the reflector (Figure 1).

**Figure 2:**
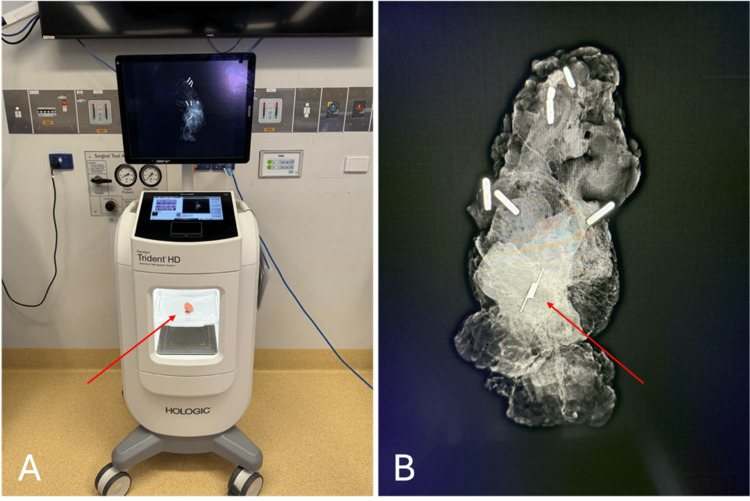
Utilisation of TRIDENT portable X-ray device intraoperatively. (A) TRIDENT machine with specimen (red arrow) used to confirm reflector in excised sample; (B) X-ray of reflector (red arrow) in resected breast lesion.

All SCOUT® reflectors were inserted by specialised breast radiologists at a single private radiology facility. Mammography was performed in all patients immediately post insertion of the reflector to confirm appropriate placement. Migration of the reflector beyond the margin of the lesion on mammography was considered unsuccessful reflector placement. All excisions were performed by one of two fellowship-trained breast surgeons who each operated at both hospitals. In the tertiary hospital, intraoperative specimen X-rays were obtained using the TRIDENT^TM^ Specimen Radiography System (Hologic, Marlborough, MA, USA) [16] to evaluate if the lesion/reflector was excised with adequate radiologic margins (Figure 2). In the private practice, the specimens were sent to the radiology facility to be X-rayed, and a radiologist provided an immediate verbal description of radiological margins to the surgeon while they continued with the operation. This prompted further intraoperative margin excision in some cases at the discretion of the surgeon based on their subjective visual assessment.

Outcomes measured

Demographic information included age, body mass index (BMI) and American Society of Anaesthesiologists (ASA) score. Diagnostic pathway, localisation imaging modality, and imaging target for localisation were also recorded. Primary outcomes were rates of successful reflector placement, localisation and retrieval, as well as positive specimen margins. Secondary outcomes included days between reflector insertion and surgery, operative duration, intraoperative re-excision, specimen weight and volume, post-operative complications within 30 days, rate of close margins, rate of re-excision, and rate of clear margins on re-excision. Tumour characteristics, including pathology, size, grade, hormone status and rates of pathologic complete response (pCR) for neoadjuvant cases, were also recorded. Positive margin was defined as ‘ink on tumour’ and close margin was defined as less than 2mm radial margin clearance for ductal carcinoma in situ. The requirement for re-excision for positive or close margins was determined by the multidisciplinary team based on the current Cancer Australia guidelines [17].

Statistics

Quantitative variables were summarized using measures of central tendency and dispersion; mean and standard deviation for normally distributed data, and median and interquartile range for non-normal data. Dichotomous data were summarized as frequencies with percentages. Statistical analyses were performed using Stata v19 (StataCorp LLC, College Station, TX, USA).

Ethics statement

This study was endorsed by the Human Research Ethics Committee (HREC) Townsville Hospital and Health Service as a clinical audit (reference number EX/2005/QTHS/122641).

## Results

A total of 170 SCOUT® reflectors were used to localise 170 nonpalpable breast lesions in 163 patients (Table 1). The mean age of the total cohort was 61.1 years (SD 12.5 years). The mean BMI was 30.0 (SD 6.5) and median ASA was 2 (IQR 2, 3). A total of 93 (55%) lesions were identified via the national breast screening program, while 52 (31%) lesions were diagnosed on imaging performed to investigate breast complaints such as a lump, nipple changes or mastalgia. The reflectors were inserted using either ultrasound or stereotactic guidance, in 146 (86%) and 24 (14%) cases, respectively. The target for localisation was either the lesion itself visualised on imaging in 151 (89%) cases, or a localising clip placed at the time of biopsy in 19 (11%) cases. Procedures performed included 39 (23%) excisional biopsies, 23 (14%) wide local excisions, 89 (52%) wide local excisions with sentinel lymph node biopsies, and 19 (11%) wide local excisions with another procedure, most commonly being an axillary dissection or oncoplastic reconstruction. A small cohort of 24 patients had their lesions excised following NACT, and the main outcomes of interest for these lesions were reported as a separate subgroup.

**Table 1:** Patient demographics and diagnostic and procedural pathway. ASA, American Society of Anaesthesiologists; BMI, body mass index; IQR, interquartile range; SLNBx, sentinel lymph node biopsy; SD, standard deviation; WLE, wide local excision.

Demographics		Whole cohort, n=170
Age, mean (SD)		61.1 (12.5)
BMI, mean (SD)		30.0 (6.5)
ASA grade, median (IQR)		2 (2, 3)
Diagnostic pathway, n (%)	Breast Screen Queensland	93 (54.7)
	Symptomatic	52 (30.6)
	Surveillance	16 (9.4)
	Incidental	9 (5.3)
Localisation modality, n (%)	US	146 (85.9)
	MMG	24 (14.1)
Imaging target for localisation, n (%)	Lesion	151 (88.8)
	Localising clip	19 (11.2)
Procedure performed, n (%)	Excisional biopsy	39 (22.9)
	WLE	23 (13.5)
	WLE + SLNBx	89 (52.4)
	WLE + other	19 (11.2)

Seven patients had two separate lesions excised, each localised by their own reflector. The remaining patients had only one lesion excised with SCOUT® localisation. The tumour characteristics of the excised lesions are summarised in Table 2. Median time from insertion of the reflector to surgery was six days (IQR 4, 13). Five patients had their SCOUT® reflectors placed on the morning of surgery. The longest duration between reflector placement and surgery was 198 days, with the reflector placed prior to the patient receiving NACT. Successful placement of the reflector into the lesion was observed in 165 (97%) cases. Four instances of unsuccessful placement were due to migration of the reflector along the insertion (needle) tract, and in one instance, the lesion moved on multiple attempts to insert the reflector, such that its final position was just beyond the margin of the lesion. The reflectors were successfully localised intraoperatively using the radar technology in 166 (98%) cases and successfully retrieved in all (100%) cases. Failed intraoperative localisation occurred twice due to inadvertent deactivation of the reflector by diathermy, and twice due to the absence of signal due to deep placement of the reflector, whereby one was placed at 40mm deep but was displaced deeper intraoperatively, and the other was placed at 60mm deep.

**Table 2:** Tumour characteristics of nonpalpable breast lesions excised with SCOUT® localisation. DCIS, ductal carcinoma in situ; ER+, oestrogen receptor positive; HER2+, human epidermal growth factor receptor 2 positive; ILC, invasive lobular carcinoma; IQR, interquartile range; mm, millimetres; NACT, neoadjuvant chemotherapy; NST, no special type; PCR, pathologic complete response; PR+, progesterone receptor positive.

Tumour characteristics		Whole cohort, n=170
Pathology, n (%)	Benign	44 (25.9)
	DCIS	24 (14.1)
	Invasive carcinoma NST	64 (37.6)
	ILC	16 (9.4)
	Tumour bed only (NACT)	12 (7.1)
	Other	10 (5.9)
Tumour size, median mm (IQR)		15 (9, 24)
Tumour grade, n (%)	1	24 (28.9)
	2	54 (65.1)
	3	5 (6.0)
Hormone status, n (%)	ER+	89 (89.9)
	PR+	82 (82.8)
	HER2+	9 (10.5)
	Triple negative	5 (5.8)
PCR, n (%)		14 (58.3)

The mean operating time was 87.5 minutes (SD 43.5). Median specimen weight was 33 grams (IQR 18, 57.8) and median specimen volume was 85.2 cubic centimetres (IQR 36.1, 136.5). After excision of the lesion and X-ray evaluation of the specimen, 32 (19%) patients had further re-excision of margins in the same procedure. Post-operative complications were observed in 14 (8%) patients. There were 12 seromas, which were all drained in an outpatient setting. Four of these had localised infection treated with antibiotics. There were two cases of haematoma, of which one required evacuation in theatre and one was aspirated in the outpatient clinic.

The number of lesions excised with involved margins was 16 (9%). An additional 22 (14%) lesions had close margins, with 33 (19%) patients requiring a secondary operation to re-excise further tissue for margin clearance. The most common method of re-excision was margin re-excision, which occurred on 26 (76%) occasions, with 7 (21%) patients proceeding directly to mastectomy. Margins were clear on re-excision in 31 (94%) cases. These surgical outcomes are summarised in Table 3.

**Table 3:** Surgical outcomes of patients undergoing SCOUT® localised excision of nonpalpable breast lesions. CC, cubic centimetres; g, grams; IQR, interquartile range; min, minutes; SD, standard deviation.

Outcomes	Whole cohort, n=170
Successful reflector placement, n (%)	165 (97.1)
Days between reflector insertion and surgery, median days (IQR)	6 (4, 13)
Successful intraoperative localisation using radar technology, n (%)	166 (97.6)
Reflector retrieval, n (%)	170 (100)
Intraoperative re-excision, n (%)	32 (18.8)
Operative duration, mean min (SD)	87.5 (43.5)
Specimen weight, median g (IQR)	33 (18, 57.8)
Specimen volume, median cc (IQR)	85.2 (36.1, 136.5)
Post-operative complications, n (%)	14 (8.2)
Involved margin, n (%)	16 (9.4)
Close margin, n (%)	22 (14.3)
Re-excision, n (%)	33 (19.4)
Clear margin on re-excision, n (%)	31 (93.9)

Each of the 24 patients who underwent surgery post-NACT had one lesion excised via SCOUT® localisation. All 24 (100%) reflectors were placed successfully and retrieved. One of the aforementioned instances of failed intraoperative localisation (due to diathermy deactivation) was in a post-NACT patient. Mean operating time was longer at 108.3min (SD 37.0). One lesion was excised with an involved margin, and one lesion was excised with a close margin. Both of these patients proceeded to margin re-excision with clear margins. These surgical outcomes are summarised in Table 4.

**Table 4:** Surgical outcomes of patients undergoing SCOUT® localised excision of nonpalpable breast lesions post-NACT. Min, minutes; SD, standard deviation.

Outcomes	NACT cohort, n=24
Successful reflector placement, n (%)	24 (100)
Successful intraoperative localisation using radar technology, n (%)	23 (95.8)
Reflector retrieval, n (%)	24 (100)
Operative duration, mean min (SD)	108.6 (37.0)
Involved margin, n (%)	1 (4.2)
Close margin, n (%)	1 (4.3)
Re-excision, n (%)	2 (8.3)

## Discussion

This study analysed the use of SCOUT® for the surgical localisation of nonpalpable breast lesions in a sample of 163 patients, with a total of 170 SCOUT® reflectors inserted. There were only five instances out of 170 (3%) where the reflector was not present within the lesion on post-insertion mammography, with migration along the insertion tract being the most common reason for imprecise placement. Three of these patients required re-excision for involved or close margins. Intraoperatively, there were four instances (2%) where the handpiece could not localise the reflector - twice due to deactivation of the reflector by diathermy, and twice due to the distance between skin and reflector being too far to elicit a signal. Further excision of breast tissue supplemented by intraoperative imaging with the Trident^TM^ allowed for successful excision of these lesions with clear margins in all but one case. Despite these complications, all reflectors were successfully retrieved in the index operation. Our results of successful reflector placement and intraoperative localisation were similar to that of other published data [12,14,18,19].

The rate of involved margins of 9% is comparable to other published data [12, 14, 20]. The published re-excision rates from other studies are somewhat variable, likely due to the lack of statistical power in studies with small cohorts and the differences in indication for re-excision. In this study, all malignant lesions with involved margins or close margins (defined by <2mm clearance of DCIS from radial margins) were re-excised, potentially leading to higher re-excision rates compared to other institutions. Nonetheless, our re-excision rate of 19% is similar to larger published studies [14, 21].

The mean operating time of 87.5 min was approximately 20 min longer than the reported mean operating times from other studies [20]. However, it is unclear how these studies measured the start and end times of the operation. In this study, operating time refers to the total time from skin preparation to dressings, and all procedure types were included in this average. Thus, this time is not an accurate reflection of the actual time taken to excise a single lesion under SCOUT® guidance alone. The mean operating time for the NACT subgroup was longer again at 108.3 min, noting that one-third of these patients had an axillary clearance in the same surgery.

In Australia, the use of the SCOUT® system has become increasingly popular, with the technology being introduced into several hospitals in Queensland. In Townsville, the SCOUT® system was selected to replace the wire localisation technique due to promising clinical data demonstrating its effectiveness for intraoperative, non-radiation-emitting, localisation of nonpalpable lesions with acceptable margin clearance [12-14, 18-21]. While not objectively measured in our data, both radiologists and surgeons have expressed their satisfaction with the usability of the SCOUT® system and satisfaction with outcomes. In our experience, the benefits of the SCOUT® system can be categorised into the pre-operative and intraoperative benefits. Pre-operatively, the reflector can be safely placed days in advance of surgery. In addition, the reflectors can be placed prior to the commencement of NACT, thereby allowing the accurate identification of the initial tumour location even if pCR is achieved. Another clinical advantage of the SCOUT® system is that the reflectors are compatible with magnetic resonance imaging (MRI), and unlike other nonwire modalities, produce clinically insignificant artefact. This allows the use of MRI as a modality for progress imaging scans to assess tumour response to NACT [13, 22].

Intraoperatively, the absence of wire placement allows the surgeon to plan a more optimal and cosmetic incision, thereby improving patient satisfaction and cosmesis [18]. In addition, the SCOUT® system also aids in improving theatre workflow and efficiency as it reduces the need for close coordination between radiology and surgical teams on the day of surgery [11]. The SCOUT® system is paired with the use of the TRIDENT^TM^ device in our high-volume facility, allowing for intraoperative confirmation of the SCOUT® reflector and assessment of margins. This allows for a timely assessment of the lesion instead of having the specimen sent to a separate room for imaging.

There were a few pitfalls of the SCOUT® system observed in this study. Diathermy deactivation by inadvertent diathermy contact with the reflector is an uncommon occurrence observed in our study and previous studies [12, 14]. Another issue observed was the failure to detect a signal from the reflector at certain depths. The manufacturer suggests a maximum depth of 6cm [15]; however, shorter depths of 4-5cm are recommended and may require using compression with the handpiece to get a signal [14]. Thus, SCOUT® localisation may not be a feasible localisation modality for some patients with large breasts and/or deep posterior lesions. The cost of the SCOUT® system is a potential disadvantage as it requires both an initial capital purchase of the console and handpieces, as well as the purchase of each single-use reflector [13]. The handpieces are easily damaged if care with sterilisation and familiarity with the cover is inadequate, creating additional costs.

The main limitations of this study include the small sample size, retrospective data retrieval and geographical limit of two co-located centres with two surgeons. As such, this reduces the generalisability of our findings to the Australian population at large. A bigger cohort of lesions removed post-NACT is required to draw any statistically meaningful comparative conclusions. The outcomes for re-excision and localisation rates will be influenced by multiple factors such as lesion size, location and pathology, as well as patient factors such as BMI and breast volume.

## Conclusions

In conclusion, the SCOUT® system is an effective and reliable method for the localisation of nonpalpable breast lesions with acceptable rates of successful insertions pre-operatively and localisation intraoperatively. A larger prospective multi-centre study would assist in confirming the generalisability of these findings. Further studies are required to compare its effectiveness with other modalities such as hookwire localisation and other wire-free localisation techniques in terms of clinical outcomes and cost-effectiveness.

## References

[1] (2026). Australian Institute of Health and Welfare. Overview of cancer in Australia, 2025. https://www.aihw.gov.au/reports/cancer/cancer-data-in-australia/contents/overview.

[2] Dunn N, Youl P, Moore J (2021). Breast-cancer mortality in screened versus unscreened women: long-term results from a population-based study in Queensland, Australia. J Med Screen.

[3] Kalager M, Haldorsen T, Bretthauer M, Hoff G, Thoresen SO, Adami HO (2009). Improved breast cancer survival following introduction of an organized mammography screening program among both screened and unscreened women: a population-based cohort study. Breast Cancer Res.

[4] Veronesi U, Luini A, Botteri E (2010). Nonpalpable breast carcinomas: long-term evaluation of 1,258 cases. Oncologist.

[5] Chu QD, Hsieh MC, Yi Y, Lyons JM, Wu XC (2022). Outcomes of breast-conserving surgery plus radiation vs mastectomy for all subtypes of early-stage breast cancer: analysis of more than 200,000 women. J Am Coll Surg.

[6] Rosenberg SM, Dominici LS, Gelber S (2020). Association of breast cancer surgery with quality of life and psychosocial well-being in young breast cancer survivors. JAMA Surg.

[7] Hanson SE, Lei X, Roubaud MS (2022). Long-term quality of life in patients with breast cancer after breast conservation vs mastectomy and reconstruction. JAMA Surg.

[8] Cooperman AM, Cook SA, Hermann RE, Esselstym CB Jr (1976). Preoperative localization of occult lesions of the breast. Surg Gynecol Obstet.

[9] Cheang E, Ha R, Thornton CM, Mango VL (2018). Innovations in image-guided preoperative breast lesion localization. Br J Radiol.

[10] Gunn J, McLaughlin S (2017). Current trends in localisation techniques for non-palpable breast lesions: making the invisible visible. Curr Breast Cancer Rep.

[11] Lin Q, Hou Q, Zhang C (2025). Innovations in the localization techniques for non-palpable breast lesions: make invisible visible. Breast.

[12] Cox CE, Garcia-Henriquez N, Glancy MJ (2016). Pilot study of a new nonradioactive surgical guidance technology for locating nonpalpable breast lesions. Ann Surg Oncol.

[13] Mango V, Ha R, Gomberawalla A, Wynn R, Feldman S (2016). Evaluation of the SAVI SCOUT surgical guidance system for localization and excision of nonpalpable breast lesions: a feasibility study. AJR Am J Roentgenol.

[14] Cox CE, Russell S, Prowler V (2016). A prospective, single arm, multi-site, clinical evaluation of a nonradioactive surgical guidance technology for the location of nonpalpable breast lesions during excision. Ann Surg Oncol.

[15] Merit Medical (2026). Merit Medical. SCOUT® radar localisation. https://www.merit.com/product/scout-radar-localization/.

[16] (2026). Hologic. Faxitron® TridentTM HD specimen radiography system. https://www.hologic.com.au/en-au/products/faxitron-trident-hd-specimen-radiography-system.

[17] (2026). Cancer Australia. Guidance for the management of early breast cancer. https://www.canceraustralia.gov.au/cancer-types/breast-cancer/guidance-early-management-breast-cancer/treatment/surgery.

[18] Kasem I, Mokbel K (2020). Savi Scout® radar localisation of non-palpable breast lesions: systematic review and pooled analysis of 842 cases. Anticancer Res.

[19] Cooper K, Allen E, Lancaster R, Woodard S (2022). From the reading room to operating room: retrospective data and pictorial review after 806 SCOUT placements. Curr Probl Diagn Radiol.

[20] Easwaralingam N, Nguyen CL, Ali F (2024). Radar localization of breast and axillary lesions with SCOUT: a prospective single institution pilot study. ANZ J Surg.

[21] Srour MK, Kim S, Amersi F, Giuliano AE, Chung A (2020). Comparison of wire localization, radioactive seed, and Savi SCOUT(®) radar for management of surgical breast disease. Breast J.

[22] Wong KW, Wong T, Chau CM (2025). Recognizing patient-related artefacts in MRI of the breasts: principles, imaging appearance, and solutions to minimize them. Br J Radiol.

